# Acute aortobiliac graft thrombosis

**DOI:** 10.1016/j.amsu.2021.102506

**Published:** 2021-06-11

**Authors:** Marco Leopardi, Annamaria Maggipinto, Gennaro Bafile

**Affiliations:** Vascular Surgery Unit, San Salvatore Hospital, University of L'Aquila, Via L. Natali 67100, L'Aquila, Italy

**Keywords:** Thrombosis, Occlusion, Aortic graft, Aortic repair, Infrarenal aortic aneurysm

## Abstract

**Introduction and Importance:**

Acute limb ischemia after infrarenal aortic repair is a rare complication, which in mainly described to appear in a short time after surgery.

The aim of this paper is to describe a case of a 66-year-old woman who presented at out attention with acute right limb ischemia, one year later an aortic repair for acute abdominal aortic aneurysm rupture. In the best of our knowledge there are no cases of sudden graft occlusion after such long time, described in literature.

**Case presentation:**

Patient presented with sudden pain and pallor in the right lower limb and subsequently same symptoms to the left lower limb. One year before she underwent an emergency repair for abdominal aortic aneurysm rupture with an aortobiliac graft. Computed Tomography Angiography (CTA) scan showed a complete occlusion of the infrarenal aorta, including aortoiliac graft, to the common bilateral iliac arteries. On the right side was also found a complete occlusion of the popliteal artery.

Emergent embolectomy of the right popliteal artery via the femoral artery was performed.

**Clinical discussion:**

CTA scan performed on third post-operative day showed the patency of infrarenal aorta and aortic portion of the grafts in presence of floating thrombus, right iliac branch patency and chronic occlusion of left iliac branch. A kinking of both graft iliac branches was evident after this CTA scan. CTA scan at one month demonstrated resolution of the thrombosis of the infrarenal aorta, complete patency of aortic portion of the graft and right iliac branch and chronic obstruction of the left common iliac artery.

**Conclusion:**

Acute limb ischemia caused by sudden graft occlusion one year later an aortic repair for acute abdominal aortic aneurysm rupture is a very rare event. Graft limbs kinking could explain acute thrombosis of the graft.

## Highlights

• Acute limb ischemia caused by sudden graft occlusion is a very rare event.
• Thromboembolism is the most frequent cause for early acute limb ischemia after AAA.
• Graft limbs kinking could explain acute thrombosis of the graft.

## 1 Introduction

Acute limb ischemia after infrarenal aortic repair is a rare complication, which in mainly described to appear in a short time after surgery [1].

The aim of this paper is to describe a case of a 66-year-old woman who presented at out attention with acute right limb ischemia, one year later an aortic repair for acute abdominal aortic aneurysm rupture. In the best of our knowledge there are no cases of sudden graft occlusion after such long time described in literature. Patient gave consent for the anonymous collection of their data on the standard consent sheet provided by our institution. This work is presented according to SCARE 2020 criteria [2].

## 2 Presentation of case

A 66-year-old woman was referred to our department for sudden onset of pain and pallor in the right lower limb and subsequently same symptoms to the left lower limb. One year before she underwent an emergency repair for abdominal aortic aneurysm rupture with an aortobiliac graft in another hospital.

The patient had a history of hypertension, BPCO and bilateral glaucoma, which were under medical treatment.

At physical examination there was no femoral pulse on the right side, hyposphigmic femoral pulse on the left side and no peripheral pulses on both sides. A Doppler Ultrasound (DUS) exam showed absence of right femoral flow and distal flow on both sides and an indirect flow on the left femoral artery.

**Fig. 1:**
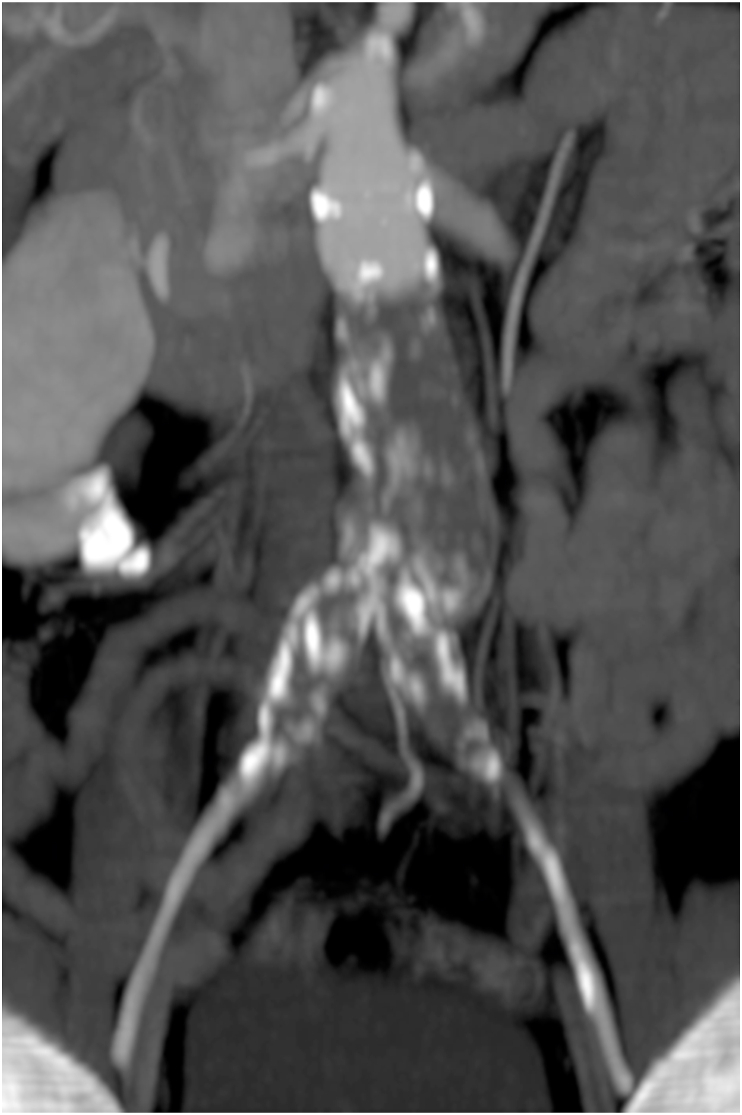
Computed tomography reconstruction of graft thrombosis.

Computed Tomography Angiography (CTA) scan showed a complete occlusion of the infrarenal aorta at 2 cm from the lowest renal artery, including aortoiliac graft, to the common bilateral iliac arteries. The internal and external iliac arteries were patent. On the right side, a complete occlusion of the popliteal artery was also found, with the presence of a late opacification of the anterior tibial artery only. Fig. 1.

Due to the acute nature of the patient's symptoms, our surgical team performed, under general anesthesia, an emergent thrombectomy of the right popliteal artery via the femoral artery. Patient was promptly informed concerning his situation and informed consensus was obtained for the intervention. Selective embolectomy with Fogarty catheters was performed on the right popliteal artery, with evidence of newly formed emboli. A proximal embolectomy of the aortic graft was also was performed, but without success. Further attempts were excluded in order to avoid graft damage, Urokinase was also injected inside aortic graft. An intraoperative angiogram confirmed the chronic nature of aortic graft occlusion and the impossibility of revascularization and good postoperative patency of the femoral-popliteal-distal axis bilaterally.

Patient was then admitted to department ward with Enoxaparin 6000 UI twice per day.

**Fig. 2:**
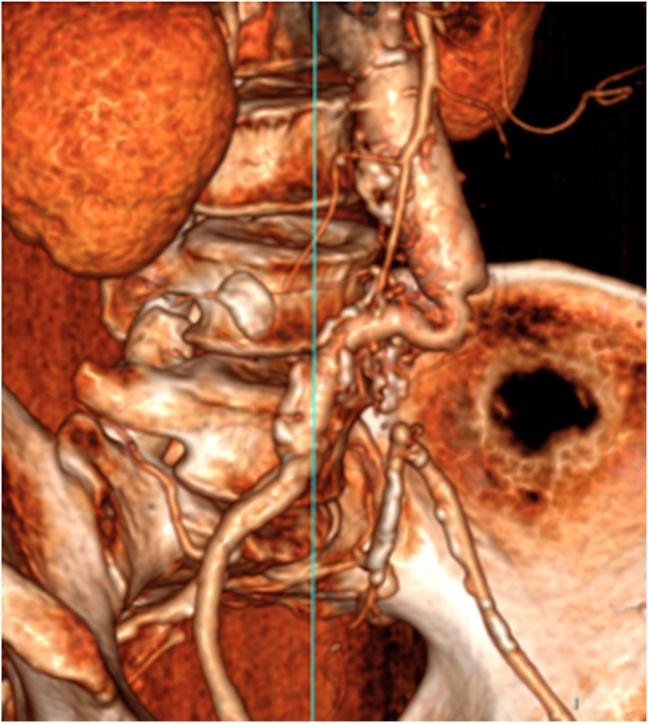
3-dimensional computed tomography reconstruction of graft recanalization.

CTA scan performed on third post-operative day showed the patency of infrarenal aorta and aortic portion of the grafts in presence of floating thrombus, right iliac branch patency and chronic occlusion of left iliac branch. A kinking of both graft iliac branches was evident after this CTA scan. Fig. 2.

The patient was discharged on eighth post-operative without complications with direct flow on right femoral artery and demodulated flow on the left femoral artery at DUS.

Patient had the first follow-up control in hospital, at one month, with a CTA scan showing resolution of the thrombosis of the infrarenal aorta, complete patency of aortic portion of the graft and right iliac branch and chronic obstruction of the left common iliac artery. Patient was then lost at follow-up.

## 3 Discussion

Aortobiliac bypass is a well-known solution for abdominal aortic aneurysms. Several complications are described and graft occlusion has been reported as less than 1% [3,4].

According to some experiences, graft occlusion is mainly described as a slow onset disease (XXX months or years) caused mainly by stenosis at anastomosis or neointimal hyperplasia [5].

Acute thrombosis with limb ischemia is a rare complication and in the best of our knowledge, there is only one case described at six months after surgery [6].

Thromboembolism is the most frequent cause for early acute limb ischemia after AAA, technical complications at the anastomoses, in situ thrombosis, and hypotension are the main causes [7].

However, rarely, limb ischemia can occur after several month of the intervention [8].

The present case describes a graft thrombosis with acute limb ischemia, which was clinically worse on the side with popliteal artery thrombosis, even if the other side was also symptomatic. Due the impossibility to obtain an easy recanalization by a supposed organized thrombus, and in order to avoid graft impairments we preferred to try a therapy with urokinase and anticoagulant. On the first postoperative day, the patient showed immediate recovery on the right side after the recanalization of popliteal artery with direct flow on popliteal artery, and slightly amelioration on the left side.

This situation leads us to suppose that the main ischemic event was represented by the popliteal artery sudden occlusion by graft thrombus embolization. But the patient amelioration with the patency of the right branch of the graft at post-operative CT scan inverted our hypothesis. Graft limbs kinking diagnosed once patency was obtained, could explain acute thrombosis of the right side and a chronic occlusion on the left side.

We could exclude stenosis at the distal anastomosis, as the right limb was completely patent after 30 days of medical therapy.

After this evaluation we could speculate that an early aortic repair with graft substitution or iliac branch regularization could prevent other occlusion in future. Anyway we can't say if this happened or not as patient was lost at follow-up.

## 4 Conclusions

Acute limb ischemia caused by sudden graft occlusion one year later an aortic repair for acute abdominal aortic aneurysm rupture is a very rare event. Graft limbs kinking could explain acute thrombosis of the graft.

## Ethical approval

Non necessary as case report.

## Author contribution

Study concept or design ML AM GB; data collection ML AM; data analysis or interpretation ML AM GB; writing the paper ML AM.

## Consent of patient

Written informed consent was obtained from the patient for publication of this case report and accompanying images. A copy of the written consent is available for review by the Editor-in-Chief of this journal on request.

## Registration of research studies

Name of the registry:

Unique Identifying number or registration ID:

Hyperlink to your specific registration (must be publicly accessible and will be checked):

## Guarantor

Marco Leopardi.

## Funding

This research did not receive any specific grant from funding agencies in the public, commercial, or not-for-profit sectors.

## Declaration of competing interest

None.
